# Gaze-Contingent Flicker Pupil Perimetry Detects Scotomas in Patients With Cerebral Visual Impairments or Glaucoma

**DOI:** 10.3389/fneur.2018.00558

**Published:** 2018-07-10

**Authors:** Marnix Naber, Carlien Roelofzen, Alessio Fracasso, Douwe P. Bergsma, Mies van Genderen, Giorgio L. Porro, Serge O. Dumoulin

**Affiliations:** ^1^Experimental Psychology, Helmholtz Institute, Utrecht University, Utrecht, Netherlands; ^2^Spinoza Centre for Neuroimaging, Royal Netherlands Academy for Arts and Sciences, Amsterdam, Netherlands; ^3^Institute of Neuroscience and Psychology, University of Glasgow, Glasgow, United Kingdom; ^4^Department of Cognitive Neuroscience, University Medical Centre St. Radboud, Nijmegen, Netherlands; ^5^Bartiméus Diagnostic Centre for complex visual disorders, Zeist, Netherlands; ^6^Ophthalmology, University Medical Center Utrecht, Utrecht, Netherlands; ^7^Experimental and Applied Psychology, VU University Amsterdam, Amsterdam, Netherlands

**Keywords:** pupillary response, perimetry, open-angle glaucoma, cerebral visual impairment, pupillometry, neuro-ophthalmic disease, visual field defect

## Abstract

**Background:** The pupillary light reflex is weaker for stimuli presented inside as compared to outside absolute scotomas. Pupillograph perimetry could thus be an objective measure of impaired visual processing. However, the diagnostic accuracy in detecting scotomas has remained unclear. We quantitatively investigated the accuracy of a novel form of pupil perimetry.

**Methods:** The new perimetry method, termed gaze-contingent flicker pupil perimetry, consists of the repetitive on, and off flickering of a bright disk (2 hz; 320 cd/m^2^; 4° diameter) on a gray background (160 cd/m^2^) for 4 seconds per stimulus location. The disk evokes continuous pupil oscillations at the same rate as its flicker frequency, and the oscillatory power of the pupil reflects visual sensitivity. We monocularly presented the disk at a total of 80 locations in the central visual field (max. 15°). The location of the flickering disk moved along with gaze to reduce confounds of eye movements (gaze-contingent paradigm). The test lasted ~5 min per eye and was performed on 7 patients with cerebral visual impairment (CVI), 8 patients with primary open angle glaucoma (age >45), and 14 healthy, age/gender-matched controls.

**Results:** For all patients, pupil oscillation power (FFT based response amplitude to flicker) was significantly weaker when the flickering disk was presented in the impaired as compared to the intact visual field (CVI: 12%, AUC = 0.73; glaucoma: 9%, AUC = 0.63). Differences in power values between impaired and intact visual fields of patients were larger than differences in power values at corresponding locations in the visual fields of the healthy control group (CVI: AUC = 0.95; glaucoma: AUC = 0.87). Pupil sensitivity maps highlighted large field scotomas and indicated the type of visual field defect (VFD) as initially diagnosed with standard automated perimetry (SAP) fairly accurately in CVI patients but less accurately in glaucoma patients.

**Conclusions:** We provide the first quantitative and objective evidence of flicker pupil perimetry's potential in detecting CVI-and glaucoma-induced VFDs. Gaze-contingent flicker pupil perimetry is a useful form of objective perimetry and results suggest it can be used to assess large VFDs with young CVI patients whom are unable to perform SAP.

## Introduction

When patients report visual impairments and an ophthalmologist suspects a visual field defect (VFD), a batch of tests will be performed. One typical test for detecting VFD is perimetry, which tests the patient's vision (i.e., visual sensitivity) across several locations of the visual field. The mostly used, standard, conventional form is threshold perimetry in which patients are shown small light points for short durations at varying light intensities and locations. Patients are asked to fixate centrally on a display and respond whenever a point in the para-fovea or periphery is seen. Point intensities and locations are adapted until the lowest visibility threshold is found for each location in the visual field.

Despite its common use, the subjective character of standard automated perimetry (SAP; also refered to as standard conventional perimetry; SCP) brings along several problems. The first problem is that very young healthy children (<5 years) (1) and most of the neurologically impaired children suffering from brain damage cannot perform SAP, since these techniques require task comprehension, full cooperation, and motoric responses (2). It is estimated that only 4% of the patients that belong to the latter group are able to perform SAP (2). Second, patients may rather easily fake visual field loss (e.g., for financial or psychological reasons) and forge SAP (3). Patients with simulated visual field loss may be subjected to extensive, time-consuming procedures, and costly medical investigations are performed until the factitiousness of the symptoms are discovered (4). The third problem is that the sensitivity and reliability of SAP can be distorted by eye-movements and fixation losses. Accurate visual processing highly depends on the focus of gaze (5, 6) and covert attention (7–10).

Pupil perimetry^1^, as an alternative method for sensory perimetry, is suggested to circumvent the problems outlined above [for reviews, see (11–13)]. Pupil perimetry consists of the measurement of the amplitude or latency of the pupillary light reflex^2^ as a measure of visual sensitivity in response to the onset of bright stimuli across several locations in the visual field. Several pupil perimetry studies propose that the visual sensitivity maps measured with pupil perimetry are *qualitatively* comparable to visual sensitivity maps from threshold perimetry [e.g., (14)]. One meta-analysis and several recent publication on multifocal pupillographic perimetry reported *quantitative* evidence for pupil perimetry's effectiveness in detecting glaucoma (13, 15–18). Some additional evidence exists in favor of the effectiveness of pupil perimetry in diagnosing patients with damage to the optic tract, to or near the lateral geniculate nucleus, or to the occipital lobes. So far, damage to the optic tract does not seem to produce reliable alterations in the pupil light reflex (11, 14, 19). Damage to or near the lateral geniculate nucleus and to the optic chiasm results in weakened pupil responses when stimuli are presented in the blind fields as compared to intact fields (14, 20–23). Damage to the occipital lobe also leads to similar effects on pupil responses to stimuli presented inside and outside the blind fields of hemianopic patients (12, 14, 19, 21, 23–28). Despite these promising results, pupil perimetry has not yet progressed to a method that is commonly applied in ophthalmology. Furthermore, the effectiveness of pupil perimetry has not yet been quantitatively assessed in CVI.

One possible explanation for the low popularity of pupil perimetry could be related to the way its diagnostic effectiveness has so far been examined. For example, pupil perimetry results of patients with post-geniculate damage are often reported in the form of qualitative comparisons of visual sensitivity maps based on subjective observations, rather than quantitative analyses of sensitivities (14, 19, 21, 27, 28). Also, previous studies either did not include healthy control populations for comparison or pupil sensitivities of healthy populations could not be dissociated from those of patients (14, 20–23, 28). Pupil perimetry has also been denoted as an impractical method because of its complexity, time-consuming nature, and poor spatial resolution (11). Lastly, when comparing pupil perimetry to SAP, it is automatically assumed that both measurement outcomes stem from the same underlying neural circuitry. However, the mechanism that determines sensitivity in pupil perimetry is not necessarily the same as the mechanism underlying visual awareness of brightness in SAP. Pupil perimetry may thus have the potential to complement SAP, independent of the type of patient, in addition to being a replacement test for patients that are unable to reliably report visual perception.

**Figure 1:**
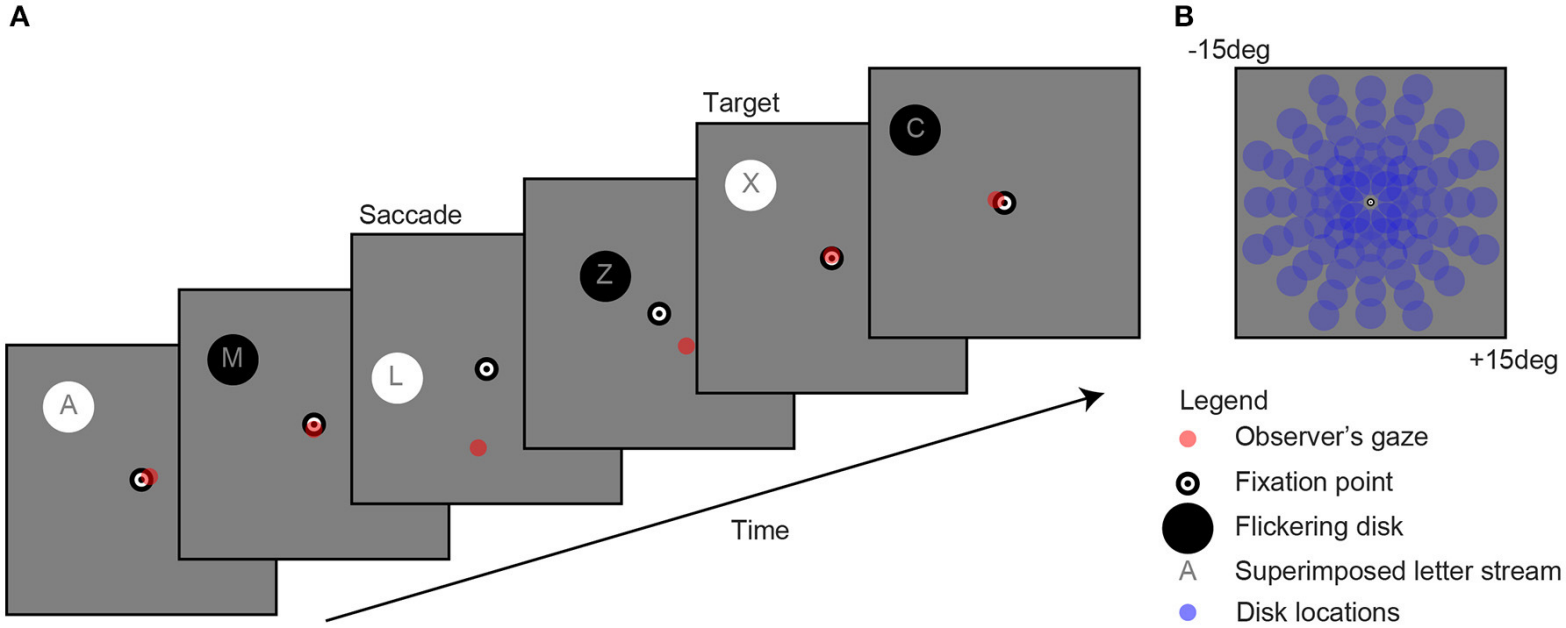
Example of a single trial **(A)**. Observers fixated a bull's eye at the center of the screen while a flickering disk was presented in the periphery at one of 80 locations **(B)** to evoke pupil oscillations. A gaze-contingent stimulus presentation ensured that the retinal location of the stimulus was fixed (e.g., see saccade). The observer's task was to attend the stream of characters on top of the disk and mentally count the number of appearances of the letter “X.” For reasons of clarity, the scaling of the stimuli in the current figure diverges from those used in the actual experiment.

The current study examines the effectiveness of pupil perimetry with a different approach than the studies described above. More specifically, we will control for effects of eye movements by (i) measuring where observers are fixating and (ii) adjusting the position of the stimulus contingent with gaze [see Figure 1; also see (29)]. Second, we ensure that patients have proficient endogenous attention for the light stimuli to evoke reliable pupil responses by superimposing a letter detection task on top of the target stimuli (7). Third, we increase the amount of pupillary measurements within a shorter time window with a novel approach: the presentation of flickering stimuli at 2 Hz that captures both the effects of response amplitude and variability in response latency in a single pupil power measure from a frequency spectrum analysis. Flicker stimuli reliably evoke phasic pupil responses (7), predominantly driven by cone- and rod-pathway (30). Fourth, we apply high resolution pupil perimetry at 80 locations with 4° diameter stimuli. Lastly, differences in pupil response amplitudes across visual field locations will be compared between patients with damage to the occipital lobe (cerebral visual impairment, CVI) or to the retina (primary open angle glaucoma; POAG) and age- and gender-matched healthy controls. As will be shown later in this paper, the novel approaches described above will lead to good to moderate detection of absolute scotomas in CVI and glaucoma patients, respectively.

## Methods

### Study design

We performed perimetry measurements on a healthy control group with fully intact visual fields and a patient group with absolute scotomas (i.e., partially damaged visual fields). The absolute scotomas were either due to brain damage in the (extra-)striate cortex or retinal damage caused by glaucoma. We performed both a *binary* (i.e., visible or not) subjective perimetry and a *continuous* (i.e., a spectrum of visibility) objective pupil perimetry. In the subjective test, observers were asked to verbally report whether or not they had seen a flickering stimulus. When the flickering stimulus was not seen, this indicated that no flicker was visible at all, suggesting that the location of an absolute (i.e., not a relative scotoma) was stimulated. On the contrary to CVI patients, glaucoma patients tend to have more relative scotomas when detected early, because it is a progressive disease with worse visibility at start to full blindness in later stages. As our subjective test measured only absolute scotomas, it was relatively conservative, indicating fewer VFDs than SAP in glaucoma patients.

The apparatus and stimuli were identical in the subjective and objective perimetry test. Observers viewed the stimuli monocularly and each eye was tested once. The outcomes of the SAP tests (Goldmann, Humphrey, or Octopus) were already available before the experiment (see Figures S1A, S2A in online Supplemental Materials).

### Patients and controls

We ran a power analysis to determine the sample size required to detect a small to medium effect size (Cohen's d: 0.2–0.6). The analysis was performed in R (https://www.r-project.org/) using the library “pwr.” We used Cohen's conventional effect sizes as a frame of reference to define the continuum of theoretical differences between our control and experimental conditions, ranging from “small” to “medium” effect sizes. Sample sizes were determined for a paired two-sample *t*-test, representing the comparisons in pupil sensitivities between the scotoma and intact locations in our patients. Three different standard deviations for the groups were chosen based on published results (0.2, 0.3, 0.4). The result of the power analysis indicates that a number of 15 patients is indicated to cover an expected effect size from small to medium (0.2 to 0.6) for a standard deviation ranging between 0.2 and 0.3.

A total of fifteen patients with absolute scotomas (eight patients with primary open angle glaucoma, age range: 48–75; seven patients with a CVI, age range: 46–77; for OCT macula thickness and brain lesions, see Figures S3, S4 in online Supplemental Materials) were tested and demographic and medical details of the fifteen patients can be found in Tables S1, S2 in the online Supplemental Materials. Other than occipital lobe damage and local retinal damage, none of the patients had neuropathy or ophthalmological diseases that affected pupil size. Glaucoma was diagnosed before the flicker pupil perimetry test with tonometry, pachymetry, fundoscopy, and visual field examination by an experienced ophthalmologist. Fourteen age-matched healthy controls (age range: 48–72) were tested and demographic and medical details can be found in Table S3. Healthy controls were asked before participation whether they had problems with vision, but were not tested for ophthalmologic diseases such as glaucoma.

All participants were Dutch with Caucasus ethnicity. All participants had normal or corrected-to-normal visual acuity. Participants were told that the goal of the experiment was to investigate a novel diagnostic procedure to test visibility across the visual field. Participants were told that the eye-tracker measured their oculomotor responses to the stimuli. The experiment conformed to the ethical principles of the Declaration of Helsinki, and was preregistered and approved by the local ethical committee of the University Medical Center Utrecht (Approval number: 09/350, addendum no 3). Patients and healthy controls received financial reimbursement for participation and travel, gave informed written consent on paper before the experiment, and were debriefed afterwards about the purpose of the experiment.

### Study objectives

The main objective of the current study was to develop an improved version of pupil perimetry that is able to diagnose absolute scotomas in patients. To achieve this goal, we examine to what degree the sensitivities of patients, as measured with pupil responses, differed between intact fields and scotomas, and whether these differences were absent in healthy controls. A second objective was to correctly indicate the type of VFD in patients (hemianopia, quadranopia, etc.) merely based on the pupil sensitivity maps.

### Apparatus and stimuli

Stimuli were generated on a Dell desktop computer with Windows 7 operating system (Microsoft, Redmond, Washington), using MatLab (Mathworks, Natick, MA, USA) and the Psychophysics toolbox extension. The LED Asus ROG swift presentation monitor (AsusTek Computer Inc., Taipei, Taiwan) displayed 1920 by 1080 pixels at a 100-Hz refresh rate. Screen width was 60 cm in width and 35 cm in height (320 cd/m^2^ maximum luminance), and the participant's viewing distance to the screen was held stable at 55 cm with a chin and forehead rest. Pupil size and gaze of one eye per test was recorded with an Eyelink 1000 eye-tracker (SR Research, Ontario, Canada; 0.5 degree accuracy of gaze location) placed 40 cm in front of the observer right under the monitor. Eye-tracker calibration consisted of a thirteen-point calibration grid, which took ~3 min per eye. One researcher helped patients locating the calibration points while a second researcher controlled the apparatus. The experiment was conducted in a darkened room without ambient light.

As shown in Figure 1, the stimuli consisted of a black and white bull's eye that served as a fixation point (0.4 degree radius), a flickering disk (2 degree radius) that was presented on a gray background (160 cd/m^2^) at one of 80 separate locations (13.5 degree maximum eccentricity) per trial, a stream of characters superimposed on the disk (font Helvetica; not “K”, “S”, “W,” and “Z” because these are too similar to the letter “X” 7, and a red point indicating gaze. Flicker rate was set at 2 Hz, which is the optimal frequency with regard to the balance between quantity (i.e., multiple responses within one second) and quality (i.e., detectability of responses) (7). The change in stimulus luminance was between black at 0.01 cd/m^2^ and white at 320 cd/m^2^ luminance. Disk and letter locations were gaze-contingent adapted, meaning that their location was moved with the exact same angle and amplitude as each tracked eye movement (see “saccade” screen in Figure 1A).

### Procedure

All patients were tested in the late morning (<11:00) or late afternoon (>15:00), except for patient P11 and P14 that were tested in the early afternoon (14:00). Healthy controls were tested on varying times of the day. After entering the lab, the left or right eye of the participant (counterbalanced) was patched with a black eye patch to ensure monocular viewing. Next, participants started with the first session: subjective perimetry. The flickering disk was shown for 2 s per location and the location of the disk was randomized across the 80 trials. The randomization order of location was the same in both eyes. Participants were asked to fixate at the bull's eye and to detect the flickering disk. A stream of letters was superimposed on the flickering disk for a purpose only relevant for block 2 (see below). In this session, participants were instructed to ignore the content of the stream of characters. After each presentation trial, participants indicated whether they saw a flickering disk or not. The response (visible or not) was recorded by the researcher by pressing the buttons “y” or “n” on the computer keyboard, and this automatically triggered the start of the next trial. Three additional catch trials with no stimulus were added at random time points to test for false positives (i.e., if a participant saw something despite absence of stimulation). After each subjective perimetry session, participants could take a short break, before starting the second session. Depending on the reaction times of the participant, testing one eye in the first, subjective session lasted ~5–10 min.

During the second session, containing an objective pupil perimetry test, participants fixated the bull's eye and were asked to detect and mentally count the appearances of a letter “X” (see “target” screen in Figure 1A). The letter task was added to prevent that the task was too boring and that no attention was paid to the stimulus, therewith suppressing pupil responses to the stimuli (7). Each disk was presented for 4 s (i.e., longer than in block 1 to increase the number of data points per trial), followed by a 1 s inter-stimulus interval with a blank screen during which patients could relax and re-orientate. Each trial was automatically started (i.e., not with a button press as in block 1). Observer's gaze location was indicated with a red dot on the screen to provide participants and the experimenters an idea of fixation accuracy. Testing one eye in the second, objective session was kept short (6 min and 40 s) for the convenience of the patients, as well as to prevent distorting effects on pupil size due to fatigue (31). The total duration of the experiment, including the eye-tracker calibration, subjective tests, and objective tests for each eye, was not shorter than 30 min and not longer than 45 min.

### Analysis

Blink periods were detected with an automated detection method in which a blink was identified when the pupil speed crossed a threshold: a speed value higher than 4 standard deviations above the mean. Missing episodes of pupil data during blink periods were interpolated with cubic interpolation. Note that the Eyelink pupil tracking system outputs pupil size in arbitrary units rather than absolute pupil diameter in millimeters. To allow comparisons across participants, pupil size was baseline corrected per trial. Slow changes in pupil size, unrelated to visual stimulation, were removed by applying a high-pass filter through the subtraction of a low-pass Butterworth filtered pupil trace (3rd order, 0.32 Hz cut-off frequency) per trial. High frequency noise in pupil size traces was additionally removed with a low-pass filter (5th order, 30 Hz cut-off frequency).

The filtered pupil traces were transformed to the frequency spectrum domain with a fast Fourier transform (FFT) and pupil oscillation power at 2 Hz was taken as the reference measurement of pupil sensitivity to a flickering stimulus per visual field location. Other measurements such as pupillary response delay (i.e., phase), coherence ratio (2 Hz pupil power divided by sum of power values across all frequencies in the estimated power spectrum), or oscillation amplitudes of fitted sinus waves to the pupil traces were also calculated but were not as accurate as the power measure. The combination of multiple measures did not improve the dissociation between patients and healthy controls. Therefore, these alternative measures are not reported in the current paper.

**Figure 2:**
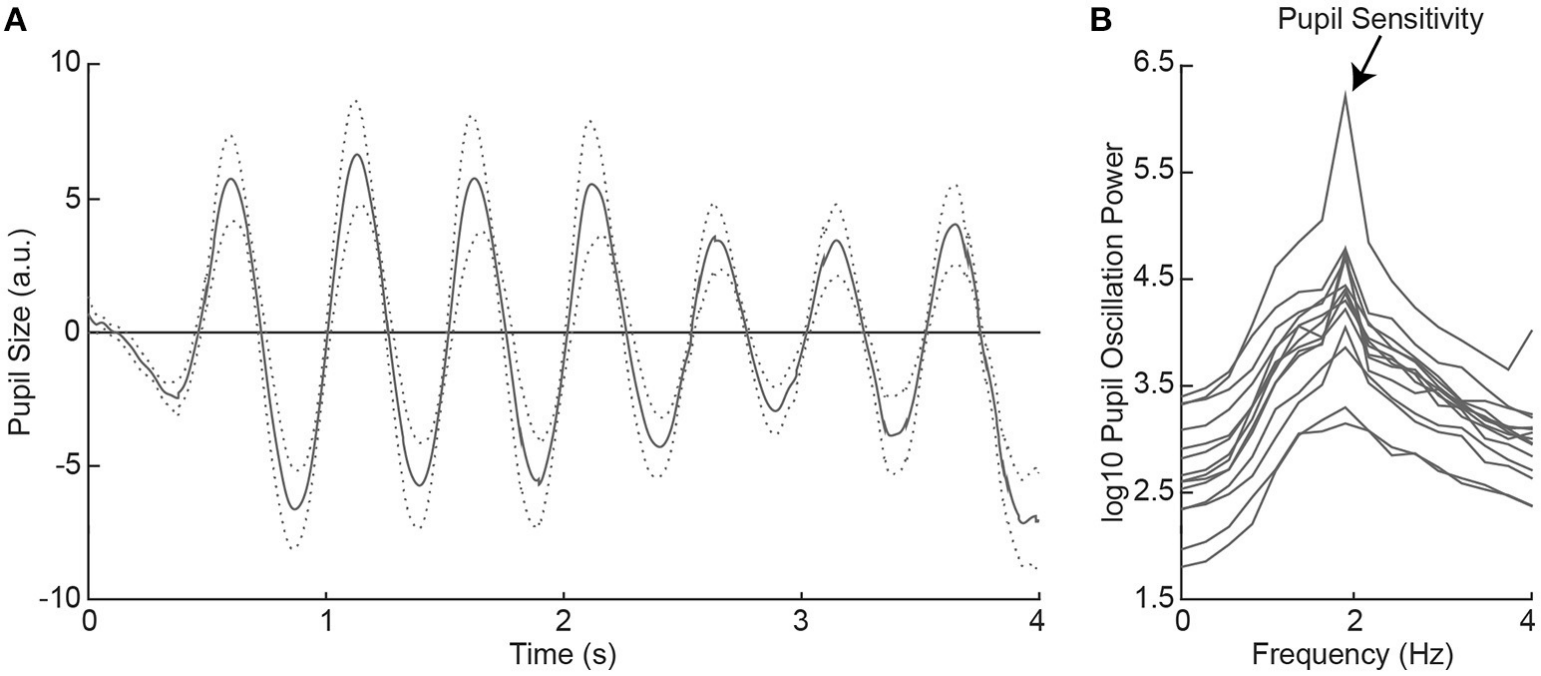
Average pupil size traces across healthy controls (HC) as a function of time during 2Hz visual flicker stimulation **(A)**. The dotted lines indicate the standard error from the mean. Pupil traces were fast Fourier transformed to a power spectrum per HC **(B)**. Pupil sensitivity per HC was based on peak pupil power at 2 Hz.

Two-dimensional high resolution pupil sensitivity maps (e.g., see Figure 2A) were created with MatLab's biharmonic spline interpolation (v4; grid data) across the 80 locations. Lastly, the most common VFDs were modeled (*n* = 10), assigning the values +1 for intact locations and −1 for damaged locations. The values in each model were multiplied with the pupil sensitivity scores of each patient. The resulting values were summed and subsequently ranked from best model overlap (#1) to lowest model overlap (#10).

Comparisons in pupil sensitivities of patients were made by calculating the area under the curve [AUC; see signal detection theory (32)] on log10 transformed pupil sensitivity distributions of the intact vs. damaged visual fields (within), or of the differences in pupil sensitivities between intact and damaged visual fields of patients vs. the differences in pupil sensitivities of the same corresponding visual fields of healthy controls. An AUC of 0.5 means that the compared distributions are fully overlapping while an AUC of 1.0 means that the compared distributions do not overlap and are fully dissociable.

Raw data of this study are available on open science framework: https://osf.io/kxqmt).

## Results

### Pupil responses to flicker stimuli in healthy controls

All healthy controls indicated to have seen the flickering disk at all locations across the visual field in the subjective perimetry test. As such, we expected that the visual stimulation with the 2 Hz flickering disk to evoke oscillatory pupil responses during the objective pupil perimetry test (7). To confirm this, we plotted the pattern of pupil responses as a function of time, first averaged across locations per participant (see Figure S5), and then averaged across participants (Figure 2A). The oscillatory pattern with two dilations and two constrictions per second (i.e., 2 Hz) was evoked in all healthy controls. Pupil sensitivity, operationalized as power at 2 Hz frequency in the fourier- and log10-transformed pupil power spectrum (Figure 2B), was on average 4.30 (*SD* = 0.59; range: 3.31–5.78). Thus, all healthy controls showed peak pupil sensitivity at a 2 Hz frequency, corresponding to the stimulus flicker.

### Pupil sensitivities in intact vs. damaged fields of patients

**Figure 3:**
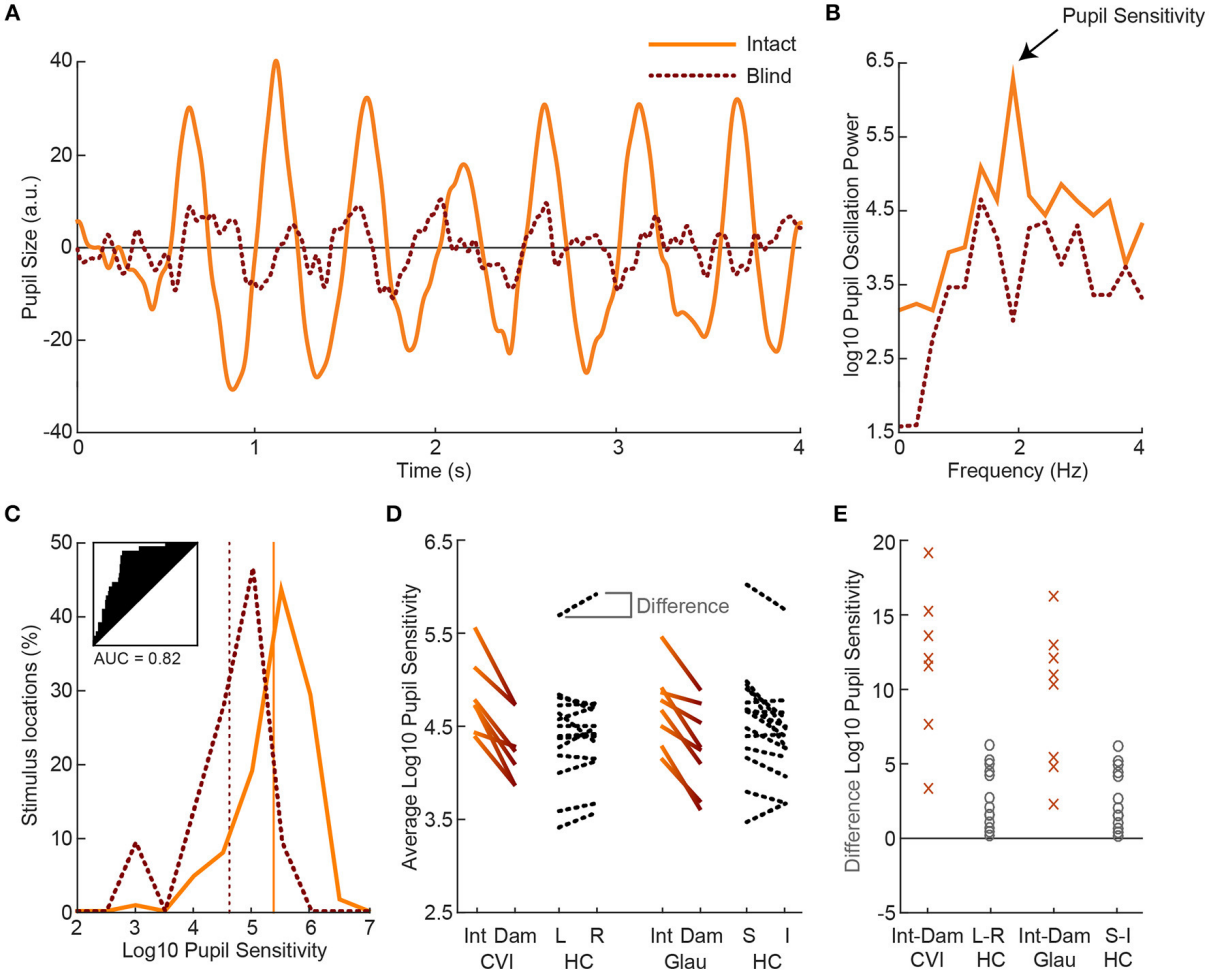
Example of pupil oscillation of the dominant eye of an exemplar patient, averaged across all stimuli presented in intact (solid) vs. damaged (dotted) visual field locations **(A)**. The traces show pupil size as a function of time during 2Hz visual flicker stimulation. Corresponding pupil oscillation power at 2 Hz as computed with an FFT are used as measure of pupil sensitivity **(B)**. The distribution of pupil sensitivities of all intact vs. damaged parts of the visual field are not overlapping (see subplot for receiver operator characteristics and AUC) for the current exemplar patient **(C)**. The average sensitivity across all intact locations and across all damaged locations are indicated with the vertical lines. Average pupil sensitivity for damaged (Dam) and intact (Int) visual fields per CVI patients (solid gradient, left) and per glaucoma (Glau) patient (solid gradient, right) as compared to average *absolute* pupil sensitivity for healthy controls (HC; dotted black) for left (L) vs. right (R) and superior (S) vs. inferior (I) visual fields **(D)**. Data were averaged across both eyes. Average percentage difference in pupil sensitivity between visual fields per CVI and glaucoma patient and per healthy control **(E)**.

Next, we examined the effect of visual field loss, as indicated by the subjective perimetry and SAP tests, on pupil responses. To determine this effect, we compared the pupil sensitivities between intact and damaged visual fields per patient. Figure 3A shows a single-trial example of pupil responses recorded from a hemianopic patient when stimulated with the flickering stimulus in an intact (solid) and damaged (dotted) location of the visual field (for average pupil responses across all intact vs. damaged regions, see Figure S6). The oscillation amplitude at 2 Hz appears stronger for the intact as compared to the damaged field, as confirmed by the power spectrum analysis (Figure 3B). We calculated pupil sensitivities for all intact and damaged locations. For the distribution of pupil sensitivities of the exemplar patient see the histogram in Figure 3C. The two distributions were reasonably separable (AUC = 0.82) with an average difference of ~0.71 log10 sensitivity between the intact and damaged visual fields. As shown in Figure 3D, the average sensitivities for damaged visual fields were weaker as compared to intact visual fields in all CVI patients (*t*_(6)_ = 6.96, *p* < 0.001) and glaucoma patients (*t*_(7)_ = 3.10, *p* = 0.017), with an average AUC of 0.73 (*SD* = 0.09) and 0.63 (*SD* = 0.10), respectively (for receiver operator curves, see Figure S7). To conclude, flicker stimulation of the damaged visual field results in lower pupil sensitivities in all patients.

### Comparisons across different types of sensitivity maps of patients

**Figure 4:**
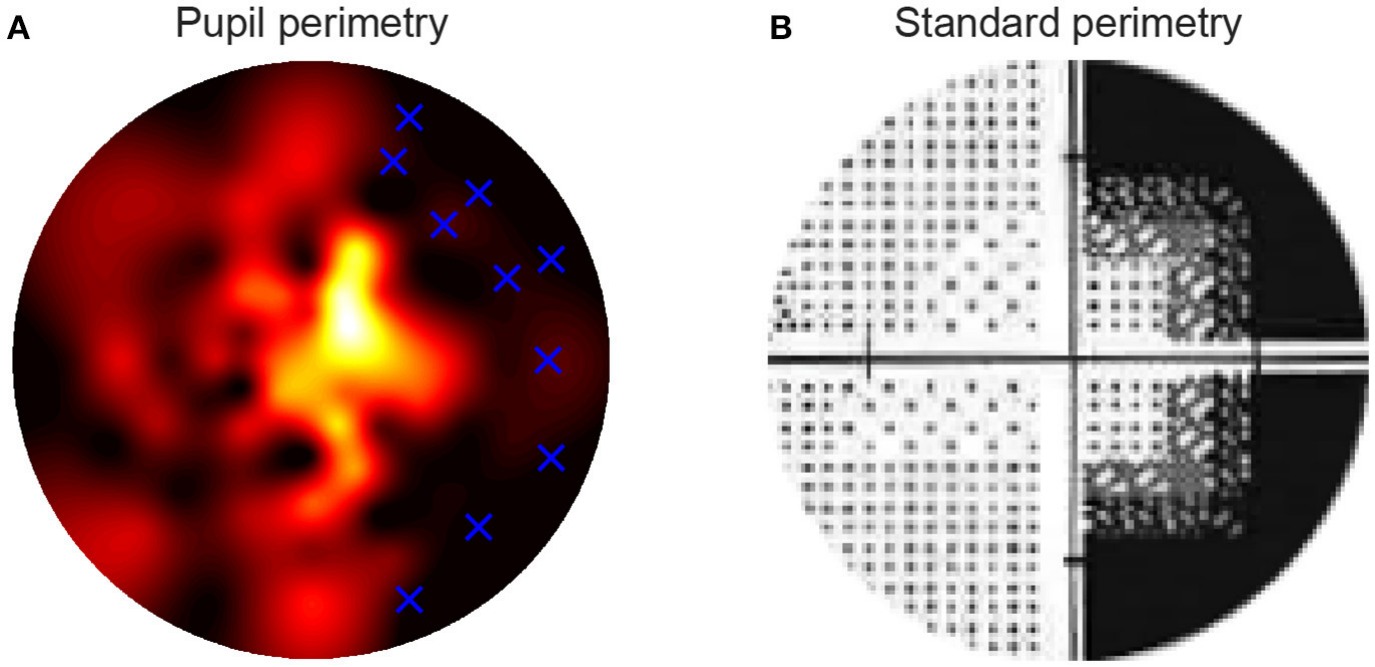
The high resolution pupillary response normalized sensitivity map was based on the pupil oscillation powers across the 80 locations in the visual fields of the right eye of a single patient **(A)**. Sensitivities were normalized from zero (weak sensitivity: red to black) to one (high sensitivity: yellow to white) per patient. The blue crosses in the map indicate the locations of the stimuli that were invisible to the patient during the subjective test in the block preceding pupil perimetry. The standard, Humphrey perimetry 30-2 sensitivity map **(B)** roughly shows similar patterns of sensitivities as in **(A)**. Note that such SAP maps normally cover a larger visual field (~30deg). The current maps only display the parts of the standard perimetry maps (<15deg radius) that were relevant for comparison with the pupil perimetry maps.

The following question that we addressed was whether the pattern of pupil sensitivities across the visual field overlapped with the pattern of SAP sensitivities and subjective visibility ratings. Two dimensional sensitivity maps per eye were already available from prior testing of patients with SAP test by ophthalmologists. Additional maps were created with the current subjective perimetry test (session 1) and objective pupil perimetry test (session 2). An example of a sensitivity map of a single patient with right hemianopia with macular sparing is shown in Figure 4. Note that the pupil responses (i.e., oscillation amplitudes) are strongest at foveal regions (i.e., close to fixation), an observation that is in line with previous pupil perimetry studies introduced before (12, 19, 28). More importantly, a qualitative inspection shows that the objective pupil perimetry, the subjective perimetry, and the SAP maps show a degree of overlap. Although the sensitivity map of pupil perimetry appears noisy on a local scale, the global visual defect on the right side of the visual field of this patient is clearly visible. Maps from other CVI patients also showed overlap globally but maps from glaucoma patients appeared to overlap less than CVI patients (see Figures S1B, S2B in online Supplemental Materials). However, qualitative inspection and comparison of these maps do not provide an objective estimate of how accurate pupil perimetry is when it comes to the detection of the type of VFDs. To get a quantitative and objective estimate of how useful pupil perimetry is as a diagnostic test, we next examined whether pupil sensitivities indeed indicated damaged visual fields.

### Dissociating patients from healthy controls

Each patient showed prominent differences in pupil sensitivities between their intact and damaged visual fields, suggesting that pupil sensitivities may serve as a diagnostic marker for problems with flicker processing. In case no prior knowledge about the patients, such as the location of damaged visual fields, is available, can pupil perimetry adequately indicate that a patient may have visual field loss and, if so, where are the scotomas located, and what type of VFD is diagnosed (e.g., hemianopia or quadrantanopia)? To answer these questions, we compared the pupil sensitivities of CVI patients vs. healthy controls and glaucoma patients vs. healthy controls with three different approaches.

First, we examined whether the average pupil sensitivities of patients, weakened by the presence of damaged visual fields, were lower as compared to the average pupil sensitivities of healthy controls. The pupil sensitivities were on average 4.82 (*SD* = 0.40) across CVI patients, 4.70 (*SD* = 0.40) across glaucoma patients, and 4.86 (*SD* = 0.43) across healthy controls (also see Figure 3D). Average pupil sensitivity did not differ between CVI patients and healthy controls (*t*_(19)_ = 0.18, *p* = 0.858) and between glaucoma patients and healthy controls (*t*_(20)_ = 0.82, *p* = 0.420). Thus, average pupil sensitivity did not dissociate patient populations from the healthy population.

Second, we assess whether patients differed from healthy controls with respect to the effect size of differences in pupil sensitivities between intact and damaged visual fields. As shown in Figures 3D,E, the difference in sensitivities between intact and damaged regions in patients were larger as compared to the differences between corresponding regions in healthy controls. Note that we separated the sensitivities of healthy controls for the left and right visual fields vs. the superior and inferior visual field to enable comparison to the CVI patients, whom all had homonymous left or right visual field hemianopia (with the exception of one patient with quadrantanopia), and the glaucoma patients, whom all had roughly superior or inferior VFDs. The percentage difference between these fields were significantly different between patients and healthy controls, and the populations were separable to a high degree (CVI: M = 11.78%, *SD* = 5.17%; HC L-R: M = 2.63%, *SD* = 2.01%; *t*_(19)_ = 5.91, *p* < 0.001, AUC = 0.95; Glaucoma: M = 9.44%, *SD* = 4.80%; HC: M = 3.57%, *SD* = 3.21%; *t*_(20)_ = 3.45, *p* = 0.003, AUC = 0.87). The difference in average pupil sensitivities of CVI vs. glaucoma patients was not significantly different (*t*_(13)_ = 0.91, *p* = 0.38).

**Figure 5:**
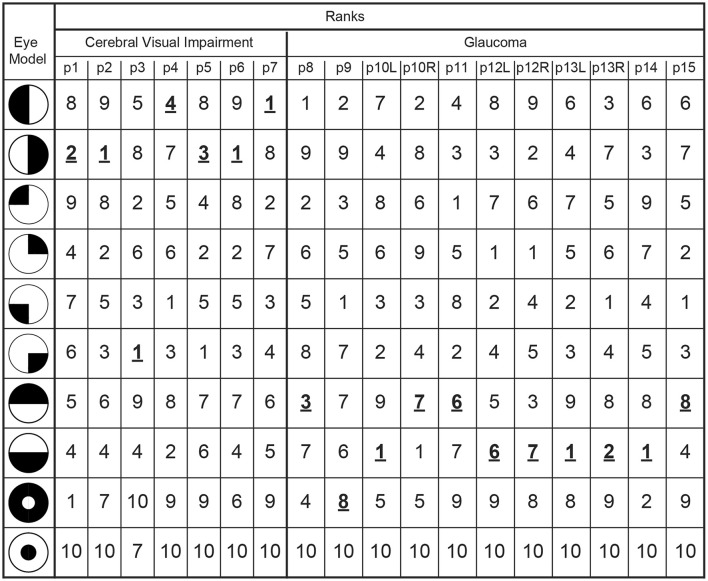
The outmost left column indicates the tested models of VFDs and the other columns lists the correlation ranks with the pupil perimetry maps per patient (CVI: p1-p7; Glaucoma: p8-p15 per left (L) or right (R) eye). The model with the highest correlation with the pupil perimetry map is ranked as number 1. The rank numbers behind the VFD models as diagnosed by SAP are printed in bold, underscored font.

The latter results imply that when pupil perimetry indicates a large difference in pupil sensitivities across certain visual fields, this could be an indication of visual field loss in an observer. However, the current study had prior knowledge about the locations of the defects (e.g., left or right visual fields in the CVI patients). Pupil perimetry only becomes a valuable test when no prior knowledge about the location of VFD is used for diagnosis. As a third analysis, we therefore compared the pupil sensitivity maps with a variety of models of VFD (left column in Figure 5) (33). The best matches between a model and a pupil sensitivity map were ranked as number one while the worst matches were ranked as number 9. A number one rank of a model means that the model most likely represents the VFD of the patient. The model representing the true visual impairment as diagnosed with SAP was ranked as number one in 4 out of 7 times for CVI patients (data was averaged across eyes) and in 3 out 11 times for glaucoma patients (Figure 5). Models that were not but should have been ranked as number one still received high ranks for CVI patients (see p1, p4, and p5 in Figure 5). Note however that patient p4 was classified as homonymous left hemianopia and that the model with the highest rank suggests inferior left quadrantanopia. However, this patient had some residual processing in the superior left visual field (see Figure S1A), perhaps explaining the higher rank for the inferior left visual field. The same applies to the right visual of patient p5. On the contrary, the ranks for glaucoma patients were less convincing, especially for patient p12 and p15.

## Discussion

We draw the following conclusions from the results: (1) the measured reduction in visual sensitivities in scotoma's measured with flicker pupil perimetry suggest that global VFDs can be detected accurately in CVI patients but less accurately in glaucoma patients, (2) absolute pupil sensitivities to stimuli presented in the blind field of patients are not dissociable from pupil sensitivities of healthy controls, but (3) the differences in pupil sensitivities between visual field locations dissociated the patients from healthy controls, and (4) the comparison of pupil sensitivity maps to predefined models of visual field loss diagnoses the type of visual defects in patients although there is room for improvement, especially for glaucoma patients. Despite the limited amount of patients tested, the current study uses quantitative analyses to demonstrate flicker pupil perimetry's potential as a diagnostic tool for CVI and glaucoma patients. The overall conclusion is that pupil perimetry sensitivities may be useful during the diagnosis and selection of the type of VFD from a set of most prevalent defects for CVI patients, but caution should be taken when interpreting such results for glaucoma patients.

When comparing the current results to previous results, a mixed picture emerges. As far as we know, no AUCs for comparisons between CVI patients and healthy controls have been reported in the literature so far. However, the high accuracy in the current paper seems to suggest that the performance of the current flicker pupil perimetry method is unprecedented. The accuracy in detecting glaucoma in the current study is also higher than several other studies (for review, see 13, although recent studies, using multifocal pupillograph perimetry, show similar results (15–18). Note, however, that these recent studies separated the AUCs for severe, moderate, and mild. When classifying the glaucoma patients tested in the current study as mild (i.e., only few of the patients had absolute scotomas in large visual field areas), the results outperform results previous studies. It is important to bear in mind though that previous studies compared the mean pupil sensitivities between patients and healthy controls, while the current study compared within-field differences in pupil sensitivities between populations. In contrast to the studies mentioned above, the mean sensitivities in the current study did not differ between populations. We can only speculate that flickering stimuli evoke pupil responses that are sensitive to within-field anisotropies, while other stimulus types (e.g., single flashes or multifocal stimuli) are better for measuring abnormalities in mean pupil sensitivities, independent of stimulus location.

Our finding that cortical damage, exclusively in the striate cortex, results in abnormal pupil responses, is causal evidence that the pupil size is at least partially driven by a cortical process in visual areas V1-V3. This has been suggested by several perimetry studies (see (12) for a review), but also has also been hinted at by several psychophysical studies [e.g., (7, 34–36), see (9) for a review]. It is thus not unlikely that both a subcortical and a cortical pathway are responsible for the pupil light reflex. A recent investigation elegantly tried to disentangle these two pathways by stimulating either the intrinsically photosensitive retinal ganglion cells (ipRGC) or the other photoreceptors using blue or red chromatic stimuli, respectively (37). CVI patients with homonymous hemianopia only showed a weakened reflex when red stimuli were presented in a scotoma as compared to an intact visual field, suggesting that the PLR evoked through blue-light-sensitive ipRGCs are subcortically controlled while the PLR evoked through the other, red-light-sensitive photoreceptors are at least partially under control of the visual cortex. The subcortical pathway is well known, and consists of the optic nerve, LGN, pretectal nucleus (and superior colliculus), Edinger-Westphal nucleus, and ciliary ganglion (38). The cortical pathway is not yet known but it is possible that the visual cortex modulates the pupil light reflex with connections to the pretectal nucleus via the LGN (39, 40).

The presence or absence of attention for the stimuli may also play a role in enhancing or inhibiting a patient's pupil responses, respectively. More attention to a stimulus is known to enhance the pupil responses to the stimulus (7–10), also during pupil perimetry (41). When patients are aware of the presentation of a stimulus presented in an intact visual field, covert attention is automatically drawn to the stimulus (normally also saccades are drawn toward the stimulus but this was inhibited by instruction to fixate), therewith enhancing pupil responses. When a patient is unaware of a stimulus presented in a damaged visual field, covert attention is not drawn, likely remains at fixation, and pupil responses are then not enhanced. A likely neural locus that may drive these attentional effects is the superior colliculus (SC). The SC is activated during the spatial allocation of attention and gaze [e.g., (42)] and recent work suggests the SC may be part of a pathway that explains residual pupil responses to a variety of unseen stimuli in humans with blindsight (43, 44), and evoked pupil responses in monkey's (45, 46).

In addition to the theoretical impact of the current findings, we provide some practical advices for ophthalmology from the patient-related results. First, we confirm that pupil perimetry cannot dissociate patients from healthy controls by merely looking at the average sensitivity across the entire visual field (47–52). However, large differences in pupil sensitivities across visual field locations (e.g., left vs. right visual field) can still be indicative of potential problems with vision, especially in CVI patients. We have taken a novel, quantitative approach to confirm that these differences allow diagnosis of visual impairments by comparing (i.e., statistically correlating) the overlap between objective pupil perimetry maps and subjective flicker perimetry maps.

Second, we can conclude from the results that scotomas by glaucoma are more difficult to detect with flicker pupil perimetry than scotomas caused by CVI. The improved accuracies in CVI patients could be due to decreased noise, as the pupil sensitivities of CVI patients were averaged across both eyes, filtering out noise. Pupil sensitivities of most glaucoma patients were only assessed per eye, because each individual eye shows a different pattern of VFDs. It is also possible that additional relative scotomas, were incorrectly classified as intact during the subjective perimetry tests, particularly for glaucoma patients. Future studies could try to circumvent such false positives by varying the contrast of the flicker stimulus. Low contrast stimuli presented at relative scotomas should then not be consciously detected by the patient. Another possibility that explains the differences across the two patient populations is that the visual sensitivity for flicker is less affected by glaucoma than the detection of faint targets in Humphrey perimetry. This interpretation is in line with our observation of a low overlap between the subjective flicker perimetry maps and the standard Humphrey threshold perimetry maps of glaucoma patients.

A general limitation of pupil perimetry in diagnosing glaucoma is described in a recent review on the effectiveness of pupil perimetry in studies with glaucoma patients (13). The authors explained that the diagnosis of glaucoma with pupil perimetry depends on comparisons of pupil sensitivities between locations in a damaged eye and the same locations in an intact eyes. Diagnosis becomes problematic when patterns of visual field losses are nearly or fully identical in both eyes, hampering comparisons of pupil sensitivities of intact and damaged regions between the eyes. Another limitation of pupil perimetry in general is that fine-grained patterns of visual field loss or small singular scotomas (<4°) will be more difficult to detect with pupil perimetry because it requires the presentation of rather small stimuli at the center of the scotoma. The main problem is that small stimuli evoke weak and unreliable (variable) pupil responses. Note that the use of very large stimuli can also be problematic due to factors such as stray light (53), although a gray rather than black background may help to suppress the influence of stray light. Furthermore, the effects of stimulus size, stimulus luminance, and background luminance (i.e., light vs. dark adaptation) on pupil responses change as a function of eccentricity [e.g., (54, 55)]. These factors may also have different effects on pupil responses than on subjective visibility reports. It will be a challenge for future studies to filter out these factors in order to measure a clean form of visual sensitivity.

One limitation of the current study is that we could only detect large scotomas. Some of the glaucoma patients had relatively small scotomas and it is possible that these went undetected because the relatively large flickering disks stimulated enough intact areas around the small scotoma to evoke a strong pupil responses. Another limitation is that one glaucoma patient (P11) took pilocarpine eye drops, which has a miotic influence on the pupils and may explain the relatively weak pupil sensitivity and diagnostic accuracy in this patient. The same patient (and P14) was tested in the early afternoon, a period known to produce less reliable SAP measurements (56). A third limitation is that, in contrast to the patients, healthy controls were not extensively tested on potential problems with vision. Although the controls reported to have no problems with vision, we do not have access to information to objectify these claims.

An interesting alternative method that also circumvents this issue is a technique that presents stimuli in maximum length sequence order [e.g., (57)], which consist of the repetitive presentation of multiple black and white patches across the visual field that independently and pseudorandomly change in luminance over time. Patches that are presented inside the area of a scotoma should then explain few of the variance in pupillary dynamics. There exists another alternative, objective perimetry method termed VEP^3^ perimetry (58, 59) that uses electrophysiological responses as measured with EEG electrodes at the scalp near occipital regions. However, VEP perimetry may not be sensitive enough to fully dissociate patients from controls (60). One solution to this challenge would be to combine VEP perimetry and pupil perimetry to improve diagnostic accuracies (11). Lastly, pupil perimetry could be improved by combining the measurement of pupil oscillations with the measurement of the post-illumination pupil response, because the latter has shown to differentiate between *early* glaucoma patients and healthy controls (61).

Future developments in flicker pupil perimetry may initiate a change in protocols in clinical practice. As mentioned in the introduction, pupil perimetry could perhaps diagnose visual field loss in neurologically impaired children and adults suffering from CVI, whom are unable to perform SAP. When these patients have relatively large scotomas, such as in CVI-induced hemianopia, pupil perimetry may detect the damaged locations. However, further studies on young and adult CVI patients, and healthy controls are necessary to confirm the possible application of pupil perimetry in a clinical setting as useful alternative for behavioral perimetry, such as the behavioral visual field test (BEFIE) (62), which has high specificity and sensitivity for absolute peripheral VFD (63). The objective character of pupil perimetry may also be utilized to confirm factitious VFDs when malingering is suspected. Lastly, the neural circuitry responsible for sensitivity in flicker pupil perimetry might be different from the neural mechanism that is responsible for visual awareness of faint targets in SAP. This means that pupil perimetry is an alternative, complementary test that may provide different insights in the type of scotoma than SAP.

One serious challenge for future work on pupil perimetry will be to ensure sustained attention to the task for at least 5 min. Patients with several cognitive deficits, caused by for example severe brain damage in multiple cortical regions, may not adhere to the task requirements. Although we ensured that patients paid attention to the visual stimulation by adding a letter detection task superimposed on the stimuli, forthcoming studies may invest in the development of stimuli that draw enough sustained attention, even from young children. Also the test's duration should be shortened while trying to maintain high data power and thus measurement reliability. The presentation of multiple stimuli at the same time in each eye may enable this (18, 19, 57, 64).

To conclude, the current study has demonstrated that flicker pupil perimetry is a promising diagnostic test for large VFDs, especially in patients with a CVI.

## Author contributions

MN, CR, AF, and SD designed the experiments. MN programmed the experiments. DB, MvG, and GP recruited patients and performed standard conventional perimetry. MN, CR, and AF collected the data. MN analyzed the data. MN wrote the first drafts of the paper and all other authors helped finalizing the final paper.

### Conflict of interest statement

The authors declare that the research was conducted in the absence of any commercial or financial relationships that could be construed as a potential conflict of interest.

## Footnotes

^1^Pupil perimetry is also termed pupillomotor campimetry or pupillograph(ic) perimetry.

^2^For readability we use the general term *response* instead of the subcomponents of a pupil response such as amplitude, latency, or phase.

^3^Also known as ERP perimetry or electroperimetry.
