# Supplementary material for: Gaze-Contingent Flicker Pupil Perimetry Detects Scotomas in Patients With Cerebral Visual Impairments or Glaucoma
Source: Front Neurol. 2018 Jul 10;9:558. doi: 10.3389/fneur.2018.00558 (PMC6048245; doi:10.3389/fneur.2018.00558)
Supplement: Supplementary file 1 [file Image_1.PDF]

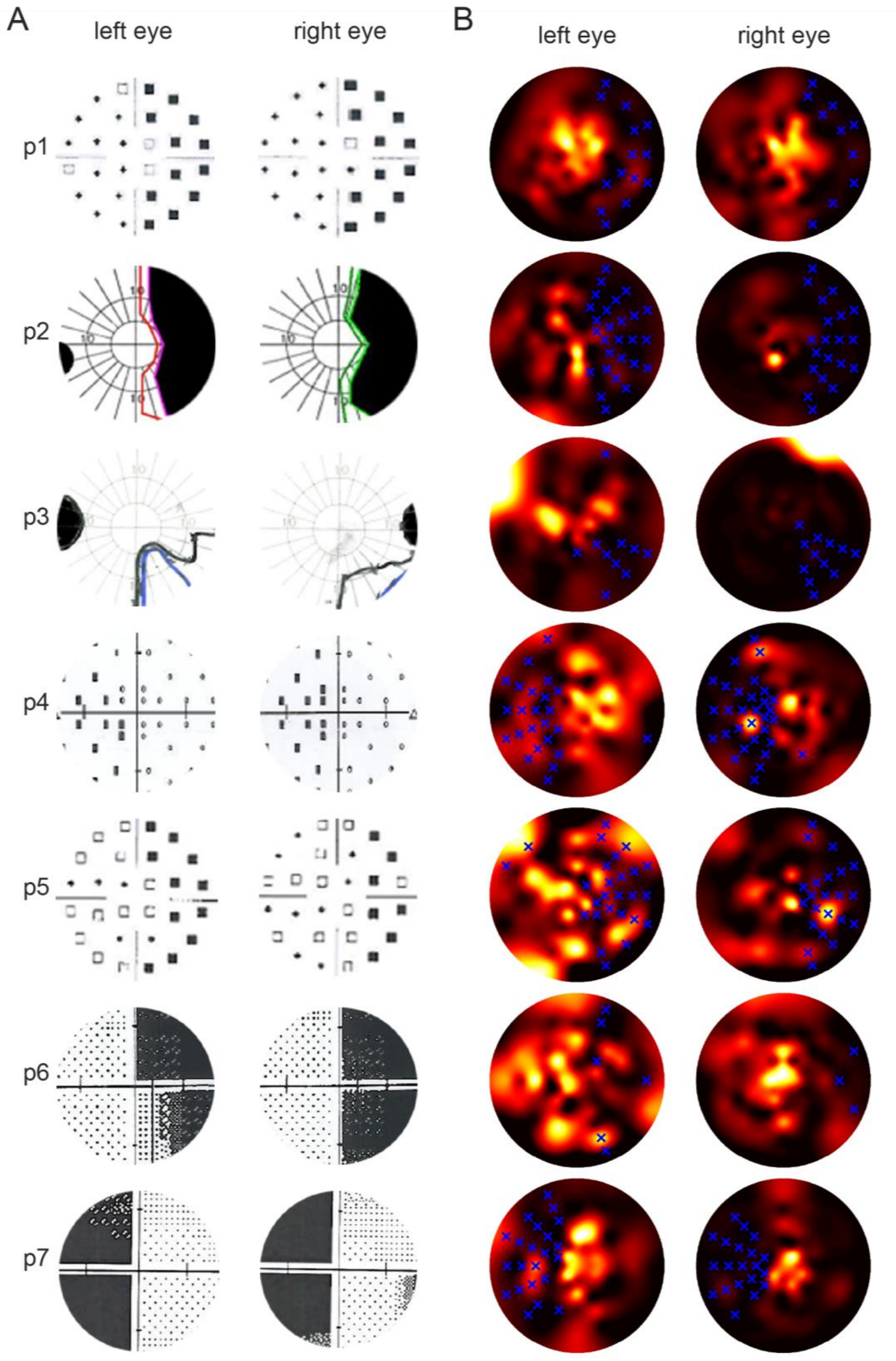

Figure S1. A, Perimetry test per CVI patient (Octopus, Goldmann, Humphrey). The visual field was cropped to an eccentricity of 15 degrees diameter to enable comparison with pupil perimetry. B, Pupil sensitivity maps per CVI patient. Yellow to white colors indicate high sensitivity while red to black colors indicate weak sensitivity. The blue crosses in the map indicate the locations of the stimuli that were invisible to the patient during the subjective flicker perimetry test in the block preceding the pupil perimetry test.
