# Supplementary figures and images for: Gaze-Contingent Flicker Pupil Perimetry Detects Scotomas in Patients With Cerebral Visual Impairments or Glaucoma
Source: Front Neurol. 2018 Jul 10;9:558. doi: 10.3389/fneur.2018.00558 (PMC6048245; doi:10.3389/fneur.2018.00558)

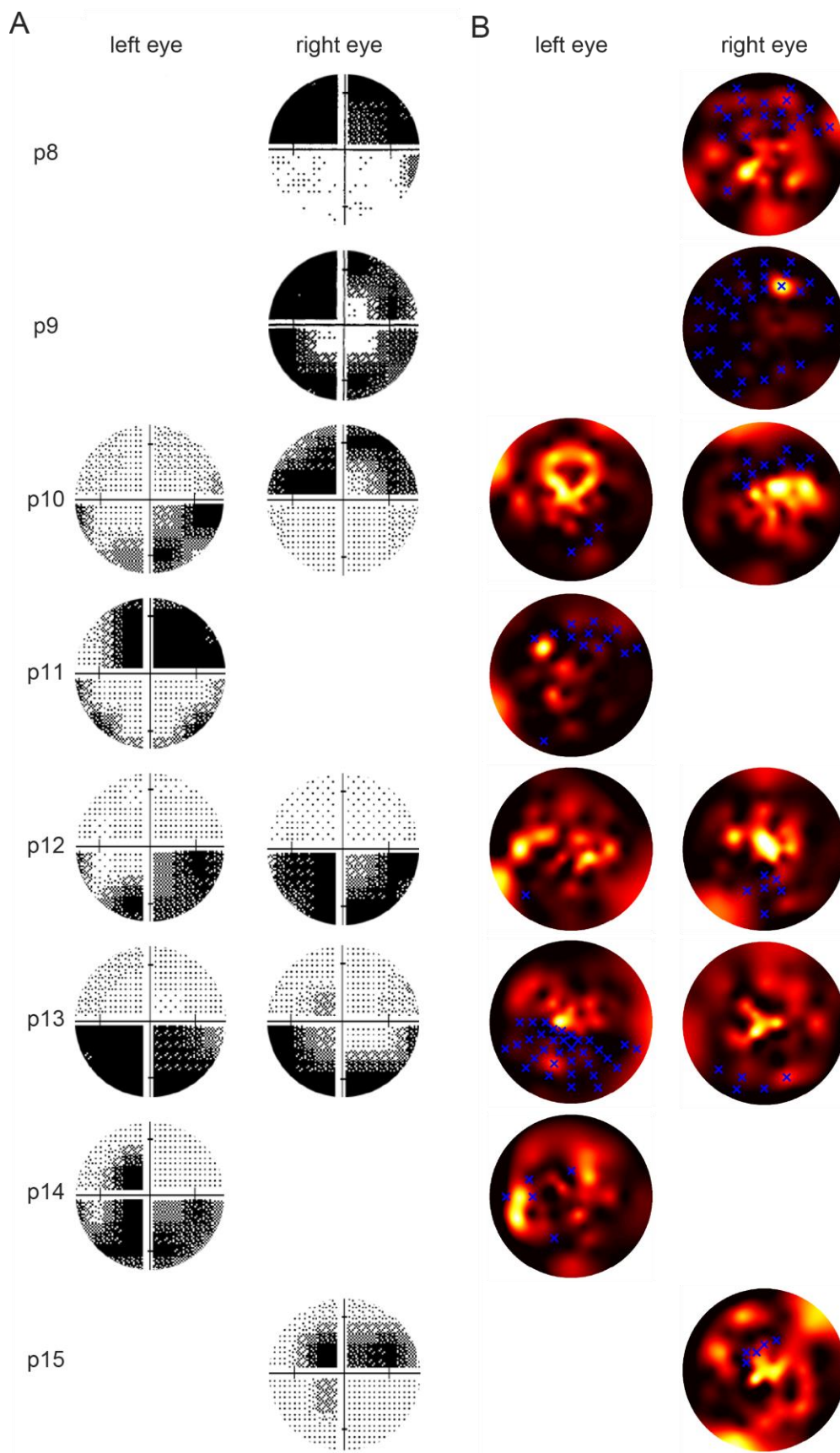

**Figure S2.** Same as Fig 1 but now for glaucoma patients.

Supplement: Supplementary file 2 [file Image_2.PDF]
