# Supplementary material for: Gaze-Contingent Flicker Pupil Perimetry Detects Scotomas in Patients With Cerebral Visual Impairments or Glaucoma
Source: Front Neurol. 2018 Jul 10;9:558. doi: 10.3389/fneur.2018.00558 (PMC6048245; doi:10.3389/fneur.2018.00558)
Supplement: Supplementary file 3 [file Image_3.PDF]

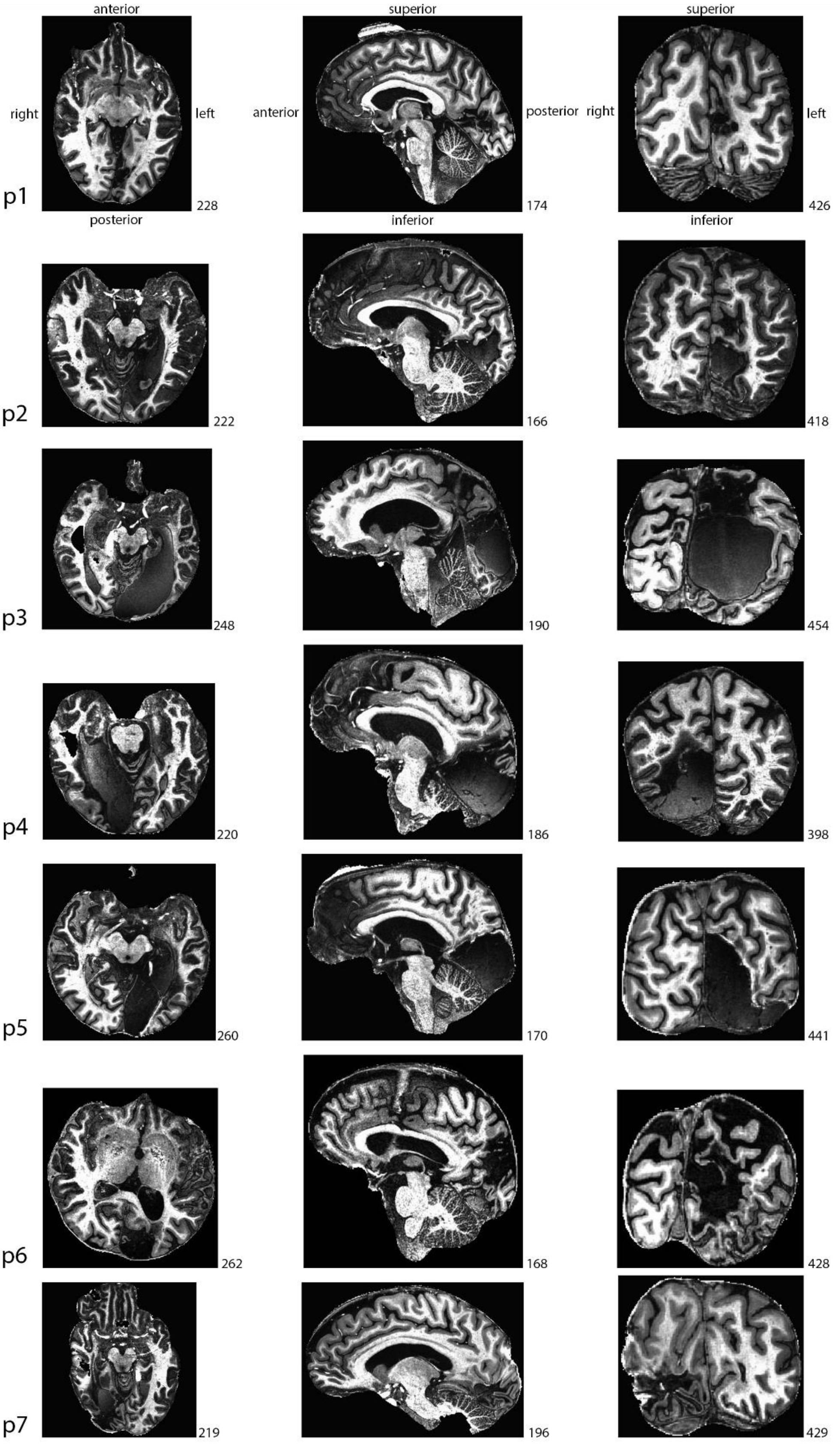

Figure S3. T1-weighted MRI scans of CVI patients, skull stripped and corrected for proton density. Left column: axial view. Middle column: sagittal view. Right column: coronal view.
