# Supplementary material for: Gaze-Contingent Flicker Pupil Perimetry Detects Scotomas in Patients With Cerebral Visual Impairments or Glaucoma
Source: Front Neurol. 2018 Jul 10;9:558. doi: 10.3389/fneur.2018.00558 (PMC6048245; doi:10.3389/fneur.2018.00558)
Supplement: Supplementary file 4 [file Image_4.PDF]

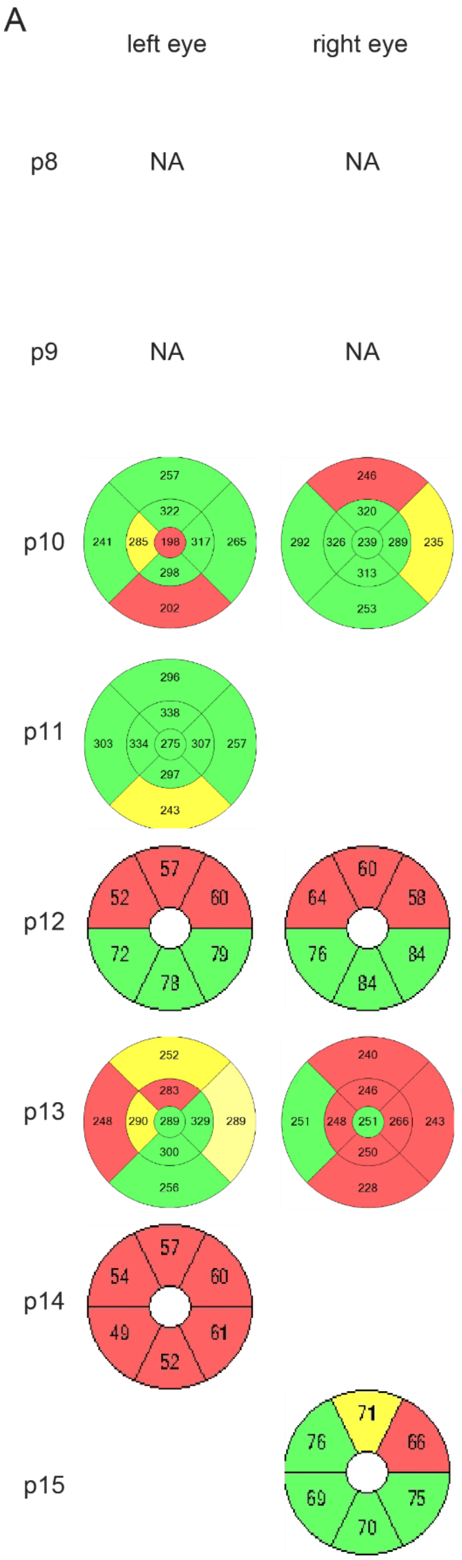

B

	left eye	right eye
p8	NA	NA
p9	NA	NA
p10	0.7	0.7
p11	0.8	
p12	0.8	0.8
p13	0.8	0.8
p14	1.0	
p15		0.8

Figure S4. A, Optical Coherence Tomography (OCT) to determine macula thickness between the Internal limiting membrance (ILM) and retinal pigment epithelium (RPE) in µm for patient p10, p11, and p13, or between the ganglion cell layer (GCL) and inner plexiform layer (IPL) for patient p12, p14, and p15. **B,** Cup-to-disc ratios per patient. OCTs and CDRs were not available for patient p8 and p9 (NA).
