# Supplementary material for: Gaze-Contingent Flicker Pupil Perimetry Detects Scotomas in Patients With Cerebral Visual Impairments or Glaucoma
Source: Front Neurol. 2018 Jul 10;9:558. doi: 10.3389/fneur.2018.00558 (PMC6048245; doi:10.3389/fneur.2018.00558)
Supplement: Supplementary file 6 [file Image_6.pdf]

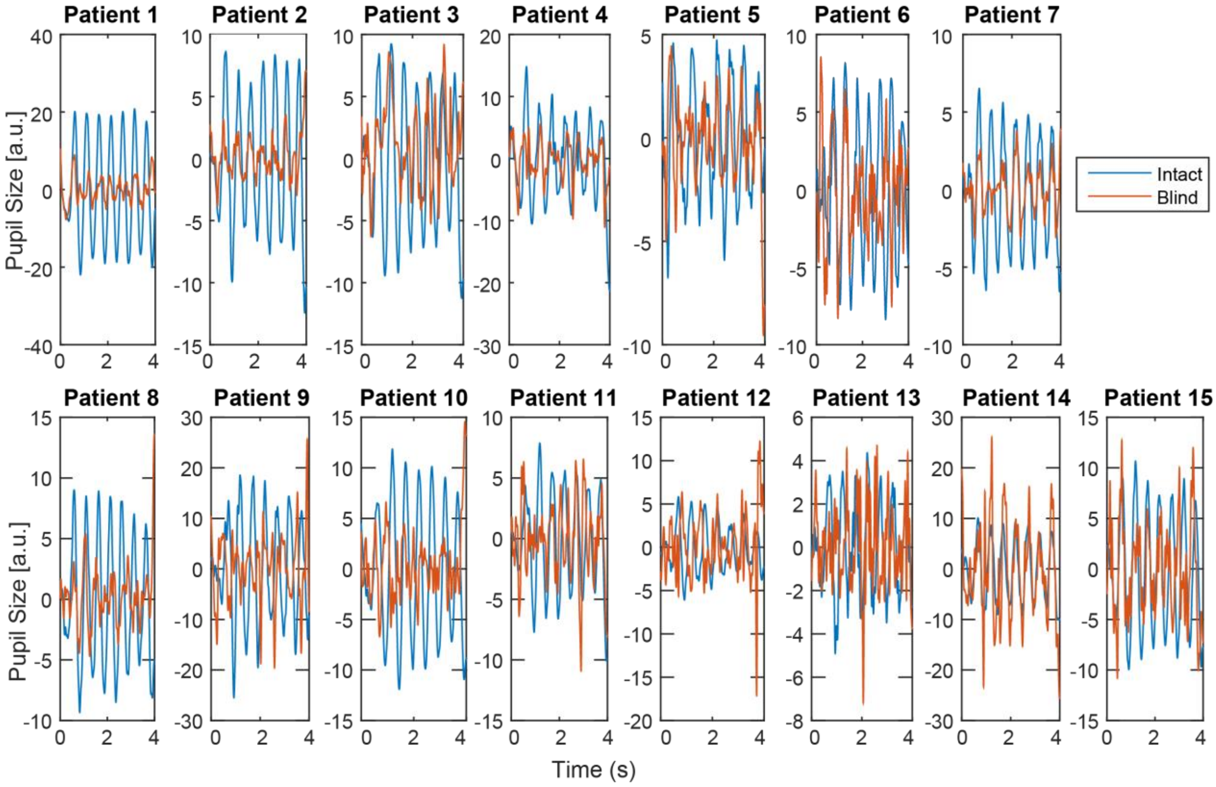

Figure S6. Average pupil responses to flicker stimuli presented in the intact (blue) versus blind (red) visual fields, per patient.
