# Supplementary material for: Gaze-Contingent Flicker Pupil Perimetry Detects Scotomas in Patients With Cerebral Visual Impairments or Glaucoma
Source: Front Neurol. 2018 Jul 10;9:558. doi: 10.3389/fneur.2018.00558 (PMC6048245; doi:10.3389/fneur.2018.00558)
Supplement: Supplementary file 7 [file Image_7.pdf]

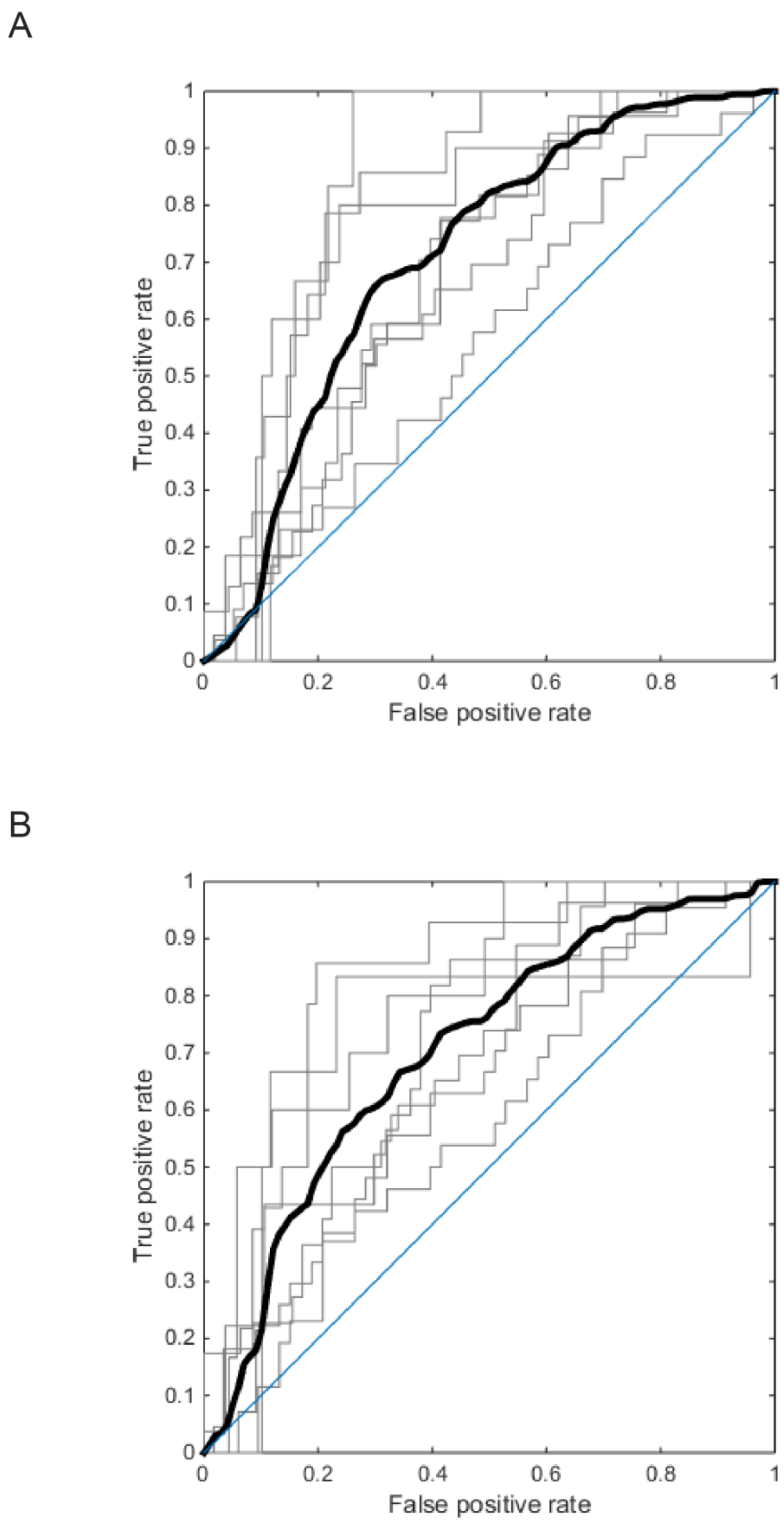

Figure S7. A, Receiver operator curves (signal detection theory) with false positive rate (x-axis) and true positive rate (y-axis) on which the AUCs were based, per CVI patient (gray) and average across all CVI patients (black). **B,** same as A but now for glaucoma patients.
