# Supplementary material for: Gaze-Contingent Flicker Pupil Perimetry Detects Scotomas in Patients With Cerebral Visual Impairments or Glaucoma
Source: Front Neurol. 2018 Jul 10;9:558. doi: 10.3389/fneur.2018.00558 (PMC6048245; doi:10.3389/fneur.2018.00558)
Supplement: Supplementary file 8 [file Table_1.PDF]

Table S1. Demographics hemianopia patients (LHH = left homonymous hemianopia. RHH = right homonymous hemianopia, IRQ = inferior right quadrantanopia).

Patient:	Age:	Gender:	Visual field defect:	Diagnosis:	Medication:
p1	46	Female	RHH	Vertebral-basilar ischemia	Simvastatin, dypirimadole, acetylsalicyl acid.
p2	60	Female	RHH	Stroke left occipital cortex	Simvastatin, acetylsalicyl acid, dypirimadole, omeprazole.
p3	60	Male	IRQ	Stroke posterior inferior cerebellar artery (PICA) left	Levetiracetam, simvastatin, amlodipine, metoprolol.
p4	65	Female	LHH	Stroke middle cerebral artery (MCA) right	Clopidogrel, hydrochlorothiazide, losartan, metformin, pantoprazole, simvastatin, spironolactone.
p5	68	Male	RHH	Stroke arterial posterior left	Pantoprazole, acetylsalicyl acid, dipyridamole, nitroglycerin, amlodipine, simvastatin, triamcinolone acetonide, salbutamol, salmeterol.
p6	77	Female	RHH	Ischemia left occipital cortex	Ascal, Allopurinol, Selokeen, Persantin, Simvastatine.
p7	63	Male	LHH	Tumor resection right occipital cortex	-
