# Supplementary material for: Gaze-Contingent Flicker Pupil Perimetry Detects Scotomas in Patients With Cerebral Visual Impairments or Glaucoma
Source: Front Neurol. 2018 Jul 10;9:558. doi: 10.3389/fneur.2018.00558 (PMC6048245; doi:10.3389/fneur.2018.00558)
Supplement: Supplementary file 9 [file Table_2.PDF]

**Table S2.** Demographics glaucoma patients. Arcuate may here also be termed altitudinal or Bjerrum scotoma. Tunnel may also be termed Tubular scotoma. Diagnosis was always POAG.

| **Patient:** | **Age:** | **Gender:** | **Visual field defect:** | **Medication:** |
|---|---|---|---|---|
| p8 | 75 | Female | LE: - RE: superior arcuate | Omeprazole, rosuvastatin, calcium vitamin D3, bimatoprost eye drops, cosopt eye drops (dorzolamine/timolol), vidisic eye drops. |
| p9 | 65 | Female | LE: inferior temporal RE: tunnel | Temazepam, trazodone, metamucil, omeprazole, artelac. eye drops (hypromellose), atorvastatin, olmesartan, cosopt eye drops (dorzolamine/timolol), bimatoprost eye drops. |
| p10 | 69 | Female | LE: inferior arcuate RE: superior arcuate | Pravastatin, desfesoterodine, brimonidine eye drops, timolol/brinzolamide eye drops, bimatoprost eye drops, carmellose eye drops, insulin, NPH insulin, metformin, acenocoumarol, hydroxocobalamin, metoprolol. |
| p11 | 56 | Female | LE: superior arcuate RE: - | Brimonidine/timolol eye drops, pilocarpine nitrate eye drops, latanoprost eye drops, hylan. |
| p12 | 65 | Male | LE: inferior arcuate RE: inferior arcuate | Anti cholesterol medication. |
| p13 | 60 | Male | LE: inferior arcuate RE: inferior arcuate | Methotrexate, alendronic acid, levoceririzine, bisoprolol, losartan, pantoprazole, folic acid, timolol, dexamethasone, hylan, fusidic acid, duratears eye drops. |
| p14 | 48 | Male | LE: inferior arcuate RE: - | Tafluprost eye drops, brimonidine eye drops, dorzolamide/timolol eye drops. |
| p15 | 60 | Female | LE: - RE: superior wedge | Metformin, brimonidine eye drops, acetazolamide, dorzolamide/timolol eye drops, bimatoprost eye drops, hylan. |
