# Supplementary material for: Gaze-Contingent Flicker Pupil Perimetry Detects Scotomas in Patients With Cerebral Visual Impairments or Glaucoma
Source: Front Neurol. 2018 Jul 10;9:558. doi: 10.3389/fneur.2018.00558 (PMC6048245; doi:10.3389/fneur.2018.00558)
Supplement: Supplementary file 10 [file Table_3.PDF]

Table S3. Demographics of healthy control subjects.

Control:	Age:	Gender:	Medication:
c1	49	Female	-
c2	57	Male	-
c3	64	Male	-
c4	56	Female	Lisinopril, hydrochlorothiazide.
c5	48	Female	
c6	53	Male	Amlodipine, atorvastatine, candesartan, pantoprazol, sotalol, xarelto, nitroglycerine.
c7	67	Female	-
c8	62	Male	-
c9	59	Female	-
c10	72	Male	-
c11	66	Male	-
c12	58	Male	-
c13	59	Female	-
c14	68	Female	-
